# Linking clinotypes to phenotypes and genotypes from laboratory test results in comprehensive physical exams

**DOI:** 10.1186/s12911-021-01387-z

**Published:** 2021-02-24

**Authors:** Thanh Nguyen, Tongbin Zhang, Geoffrey Fox, Sisi Zeng, Ni Cao, Chuandi Pan, Jake Y. Chen

**Affiliations:** 1 Informatics Institute, School of Medicine, The University of Alabama at Birmingham, AL Birmingham, USA; 2 School of First Clinical Medical Sciences - School of Information and Engineering, Wenzhou Medical University, Zhejiang, China; 3 Department of Computer Technology and Information Management, The First Affiliated Hospital of Wenzhou Medical University, Zhejiang, China; 4 School of Informatics, Computing, and Engineering, Indiana University, Bloomington, IN USA

**Keywords:** Clinotype, Lab test result, Electronic medical record, Machine learning

## Abstract

**Background:**

In this work, we aimed to demonstrate how to utilize the lab test results and other clinical information to support precision medicine research and clinical decisions on complex diseases, with the support of electronic medical record facilities. We defined “clinotypes” as clinical information that could be observed and measured objectively using biomedical instruments. From well-known ‘omic’ problem definitions, we defined problems using clinotype information, including stratifying patients—identifying interested sub cohorts for future studies, mining significant associations between clinotypes and specific phenotypes-diseases, and discovering potential linkages between clinotype and genomic information. We solved these problems by integrating public omic databases and applying advanced machine learning and visual analytic techniques on two-year health exam records from a large population of healthy southern Chinese individuals (size n = 91,354). When developing the solution, we carefully addressed the missing information, imbalance and non-uniformed data annotation issues.

**Results:**

We organized the techniques and solutions to address the problems and issues above into CPA framework (Clinotype Prediction and Association-finding). At the data preprocessing step, we handled the missing value issue with predicted accuracy of 0.760. We curated 12,635 clinotype-gene associations. We found 147 Associations between 147 chronic diseases-phenotype and clinotypes, which improved the disease predictive performance to AUC (average) of 0.967. We mined 182 significant clinotype-clinotype associations among 69 clinotypes.

**Conclusions:**

Our results showed strong potential connectivity between the omics information and the clinical lab test information. The results further emphasized the needs to utilize and integrate the clinical information, especially the lab test results, in future PheWas and omic studies. Furthermore, it showed that the clinotype information could initiate an alternative research direction and serve as an independent field of data to support the well-known ‘phenome’ and ‘genome’ researches.

## Background

As electronic health records (EHR) has been increasingly supporting biomedical and healthcare service research, utilizing the clinical information, especially the clinical test information, to strengthen precision medicine is still an open challenge [1]. Here, we have seen many EHR applications in improving precision medicine and quality of care, including: identifying disease risk factors [2], molecular biomarkers [3]; identifying high-risk/special-treatment cohorts [4, 5]; identifying the comorbidities[6, 7]; detecting drug adverse events and side effects [8]; repurposing drugs [9]; and predicting early hospitalizations [10]. However, it is still unclear to what extent the findings associate to specific clinical test results, which are among the most practical information for the care providers [11]. In addition, whether these associations imply that the test results are risk factors or just the reflection of the phenotype is still ambiguous. For example, the monocyte count, which is a popular blood test, is the result of the inflammatory response in chronic obstructive pulmonary disease and could be as a risk factor leading to cardiovascular diseases [12].

In the other hands, electronic medical data systems and analytical methods, which are the essential facilities to tackle the challenge above, have been gradually matured. At the data system component, elements in EHR data, including the medical test information, unified medical language system [13], and data integration [14] have been standardized [15–17] and well-supported to EHR extraction and refinement. In addition, from natural language processing tools [18], manual curation and crowd-sourcing efforts, there have been many data sources [19–21] potentially allows linking the clinical test results, the phenotypic/clinical outcomes, and genotype information. At the analytical component, custom statistical data mining and machine learning techniques have been applied to EHR data to cope with challenges in understanding biomedical and healthcare big data. To determine disease risks, one can use a popular statistical analysis technique—disproportionality analysis [22]. To predict patient survival and track disease progression using clinical biomarkers [23, 24], one can perform temporal data analysis such as regression in time series analysis [25] and Cox regression model [26]. To perform classifications based on multivariate models [27], one can build statistical learning models such as decision tree [28], artificial neural network [29], hidden Markov model, and support vector machine [30, 31]. In addition, set-based statistical analysis methods, such as chi-square and Fisher’s exact test are also useful in evaluating the significance of the findings [32]. There have been several examples of informatics systems allowing utilization of medical test and other clinical information, such as eMERGE [33] and I2B2 [34], where the integration of test results and genotype information would help in specifying the cohorts of interest and customized algorithm are developed for disease-specific problems.

Given these better facilities, why EHR and its rich clinical test information has not been able to play a more active role in precision medicine? Among many limitations, [35] highlights the data quality issues: “interoperability, poor quality, and accuracy of the collected information”. In other words, EHR data have has three specific challenging issues to address. First, EHR data contains missing values [36] because of human error or non-response subjects [37]. Second, EHR data is naturally imbalanced: class imbalance, for example, the small percentage of ‘abnormality’ events, and patient demographic imbalance. Third, EHR data lacks thorough and uniform annotation. Usually, the annotation needs to be made patient-specific.

This work is a pioneering framework in better-utilizing EHR, especially its rich clinical test result, to enhance precision medicine, defining new problems and providing solutions in biomedicine involving these data. We proposed the concept “clinotype” in response to the call for clinical information modeling, especially for querying and analytics over clinical content and decision support over clinical content [38]. We define “clinotypes” as clinical information, excluding the treatment, that can be observed and measured objectively using biomedical instruments. Most of the clinotypes are hospital lab tests. However, we argue that the “clinotype” concept and the “hospital lab test” are not entirely the same due to two reasons. First, with the development of mobile devices, the patients can self-perform some measurements outside the hospital laboratory; therefore, the term “hospital lab test” may not be well-applied in this case. Second, hospital lab tests include drug testing (treatment-related); therefore, this type of lab test is excluded from “clinotype” definition. In addition, different from “phenotypes” commonly used in biomedicine, which is associated to disease morphology developed by healthcare professionals [39], clinotypes are qualitative or quantitative measurements that are neutral to expert judgment. We tackled the data quality issues by both data quality control and machine learning support. We defined three board problems of ‘clinotype’ data analytics: clinotype-clinotype association discovery, clinotype-phenotype association discovery and clinotype-genotype relationship discovery. We named the framework CPA (Clinotype Prediction and Association-finding). The dataset used in this study, provided by the 1st affiliated hospital—Wenzhou Medical University—China (acronym: 1AH), contains values of totally 400 clinotypes, with no specification on interested cohorts or diseases. This dataset was collected between 2012 and 2014 from 91,354 patients, which well-represents the Southern Chinese population, mostly from south of Fujian province and the entire Zhejiang province with more than 20 million civilians.

## Materials and methods

**Fig. 1:**
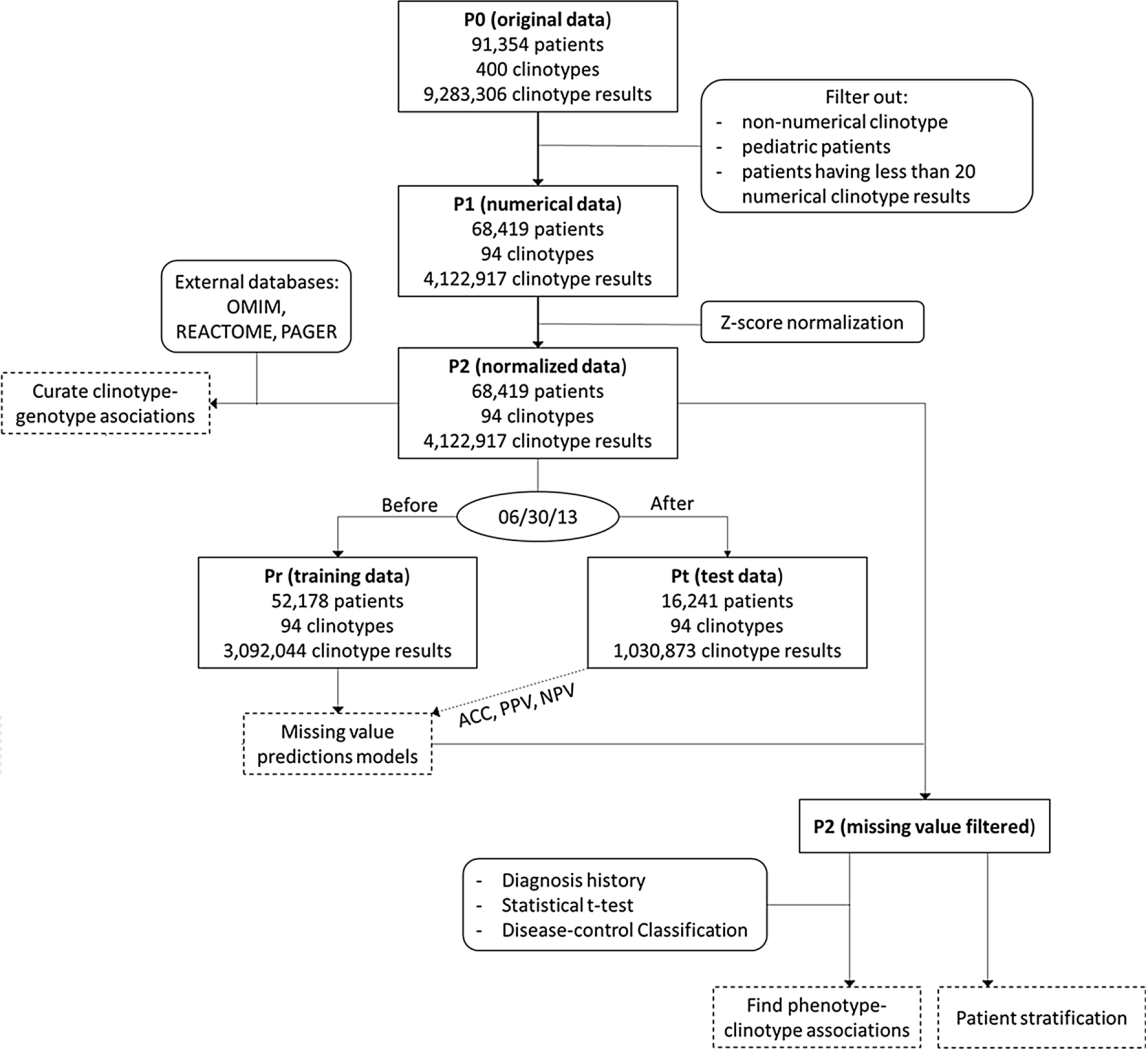
Flowchart for CPA framework. The rectangle boxes represent clinotype data subsets from P0 to Pr/Pt. The dash rectangle boxes represent clinotype problems and main results. The rounded rectangle boxes represent external (non-clinotype) data and techniques help solving the clinotype problems

CPA is an integrative machine learning framework, including data preprocessing and clinotype analysis as presented in Fig. 1. From the original data (P0), which consist of 9,283,306 clinotype results from 91,354 patients and 400 clinotypes, we filtered insignificant clinotypes and patients and normalized the data. In data preprocessing, due to technical limitations in Chinese natural language processing, we were unable to include the non-numerical clinotype results. After preprocessing, we used P2 data subset and available diagnosis information to solve the clinotypes problem: discovering clinotype-phenotype (disease) associations and stratifying the patients' clinotype data for interested cohort identification. We curated the existing 'omic' data sources for clinotype-genotype information.

### Acquire and preprocess data

We acquired, preprocessed and organized the dataset according to the workflow in Fig. 1 by 3 steps, which creates 5 data subsets: P0, P1, P2, Pr and Pt. P0 stands for the original dataset after removing patients’ identifiable information. P1 stands for subsets of data related to numerical clinotype. P2 stands for the normalized dataset from P1. Pr and Pt stand for the training set and the test set correspondingly in machine learning. The data preprocessing would tackle the non-uniform annotation issues and support machine learning as follow.

The original P0 subset, acquired directly from the health checkup (which is an independent department at 1AH), contains records on 400 health clinotype values of 91,354 patients between September 2011 and May 2014. Among 91,354 patients, 712 patients (0.7%) are under 18 years old. More information about the selected cohort could be found in Table 1. Since this work focuses on health clinotype, we manually translated the clinotype names from Chinese to English. To improve the quality of our translation, we queried our translated English name in popular medical terminology resources: MedLinePlus (http://www.nlm.nih.gov/medlineplus/), Lab Tests Online (https://labtestsonline.org/), PubMed (http://www.ncbi.nlm.nih.gov/pubmed/ for title/abstract) and adjusted our translation according to the closest matched terms in these resources. Importantly, for each personal clinotype result in P0, the 1AH provided the normal reference ranges, which referred to Chinese medical guidance and was the standard requirement at any 1AH medical record. The reference ranges are subjected to individuals. For example, the Hematocrit test in P0 has two reference ranges: 35–45% for female individuals and 40–50% for male individuals. The normal reference ranges allow annotating all clinotype results as ‘high’, ‘normal’ and ‘low’. Therefore, in this work, we tackled the annotation issue by applying the domain knowledge and data standard from the care provider.

**Table 1:** Statistics about the demographic information in the selected cohort

Age group	Gender	No. patient (%)
Young (18–39)	Male	14,594 (21.33)
	Female	12,596 (18.41)
Middle (40–59)	Male	18,717 (27.36)
	Female	14,137 (20.66)
Old (60 and above)	Male	5207 (7.61)
	Female	3168 (4.63)

The P1 subset results from P0 by filtering out low-confidence patient and clinotype information. Among 400 clinotypes, 97 clinotypes are numerical. In this work, due to the technical limitation in Chinese natural language processing, we did not include the non-numerical test result, which often include free text. Three clinotypes: Yeast Culture, Creatinine (Enzymatic) and Thyroid Globulin Antibody (ECLIA) are rare (taken by less than 1000 patients, or 1% of the population size) and excluded from the study to reduce the noisy effect in statistical machine learning methods. Thus, 94 clinotypes remained for further preprocessing and analysis. We also removed patients having no numerical clinotypes and 213 pediatric patients (< 0.1%) due to low count. P1 contains 4,122,917 patients’ health clinotypes entries from 68,419 patients.

The P2 subset results from P1 by normalizing clinotype results with the z-score formula

1 $${\widetilde{{x_{i,n}}}} = \frac{{{x_{i,n}} - {\overline{x_i}}}}{{\sigma_i}}$$

in which *i* is the clinotype index, *n* is the patient index, ${\overline{x_i}}$ is the mean of clinotype *i*,${\sigma_i}$ is the standard deviation of clinotype *i* and ${\widetilde{{x_{i,n}}}}$ is the normalized value of patient *n* on clinotype *i*. The mean and standard deviation was calculated only from the training set. We chose z-score normalization because it could remove all of the clinotype biases and variances in machine learning. In addition, z-score normalization is a linear method, which is suitable for interpreting and validating the results from linear regression later. We scaled the normal range for each individual clinotype result using the same mean and standard deviation at (1).

We setup the training subset Pr and subset Pt for downstream machine learning analysis and validation. We selected the date June 30 2013 to separate the dataset. This date divides the P2 set into a training set and test set following conventional ratio 3:1 (Fig. 1). Pt and Pr allow tackling the missing value issues using machine learning, which we would describe later. For missing values existing in Pt and Pr, we replaced them with the corresponding predicted values computed from the missing value models. The P2, Pt and Pr subsets allow defining and solving the clinotype—related problems as shown in Fig. 1 pipeline.

In addition to the P0 dataset, the outpatient department at 1AH provided the diagnostic history, identified by Chinese ICD version 10. More information about disease-specific cohort could be found in Additional file 1: Table S1.

### Handle the missing value and data imbalance

#### Technical solution

Built upon machine learning techniques, the CPA framework handled the missing value issue and partially data imbalance issue in one step. We select the support vector linear regression (SVLR) to *build models predicting the missing value*. Compared to other techniques in handling missing data [43, 44], we preferred SVLR because of not only its higher sparsity [45, 46] but also its models could be directly applied to discover clinotype-clinotype associations. For each clinotype *y*, the SVLR estimate the missing value using the linear model ${\widetilde{y_n}} = {{\mathbf{w}}^T}{{\mathbf{x}}_n} + b$ if the clinotype value of patient *n* is missing. Here, ${\widetilde{y_n}}$ denotes the estimation for missing value, **x**_*n*_ is the vector of other (non-missing) clinotype value for patient *n*, and **w** denotes the coefficient for these non-missing clinotypes. SVLR uses the non-missing *y* in Pr subset to train the model. Briefly, the SVLR setup the solution minimizing:

$$\frac{1}{2}\left| {\mathbf{w}} \right| + C\mathop \sum \limits_n^N {\xi_n}$$

2 $$\begin{aligned} & {\text{subject to}}\;\left\{ {\begin{array}{*{20}{c}} {{{\mathbf{w}}^T}{{\mathbf{x}}_n} + b \geqslant {y_n} - \varepsilon - {\xi_n}} \\ {{{\mathbf{w}}^T}{{\mathbf{x}}_n} + b \leqslant {y_n} + \varepsilon + {\xi_n}} \end{array}} \right. \\ & \quad \quad {\text{and}}\;{\xi_n} \geqslant 0\;\;\forall n \\ \end{aligned}$$

Here, *y*_*n*_ denotes the non-missing value for *y* in training, ε ≥ 0 is the ‘tolerance’, or expected error between the predicted and the real *y*_*n*_ in regression, and ${\xi_n}$ is the slack variable as defined in [45, 46]. Parameter *C* and *ε* decide the trade-off between the smoothness of regression function and how tolerance the predicted clinotype value could deviate from the true clinotype value. We decided to use C = 1 and *ε* = 0.001 after testing multiple choices of C = 0.001, 0.01, 0.1, 1, 100, 1000 and multiple choices of *ε* = 0.001, *ε* = 0.01, *ε* = 0.1 *ε* = 1. We used ILOG CPLEX Optimizer [47] to solve the problem (2).

To partially *tackle the data imbalance issue*, in implementation, we applied the under-resampling method in [48] to select the balanced subset in the training phase. By balancing, we mean for each predicted-target clinotype *y* in (2), the ratio among ‘normal’, ‘high’ and ‘low’ *y*_*n*_ selected in training is relatively 1:1:1. For each clinotype prediction, we ran resampling, learning and predicting 50 times and reported the average for coefficients and predicted value.

#### Performance metric and validation

We used the models (2) built upon Pr subset to estimate the non-missing clinotype values in Pt set. Since each non-missing clinotype value has a reference range, the real and estimated clinotype value could be annotated as either ‘high’, ‘normal’ or ‘low’. Therefore, we have 9 possible outcomes as shown in Table 2.

**Table 2:** Confusion matrix between the estimated and real clinotype value annotation

		Estimated value annotation		
		High	Normal	Low
Real value annotation	High	TP	FN	FP
	Normal	FP	TN	FP
	Low	FP	FN	TP

TP: true positive, TN: true negative, FP: false positive, FN: false negative

With the emphasize on predicting abnormality, we had the accuracy (ACC) and positive predictive value (PPV) metrics as

$${\text{ACC}} = \frac{{{\rm{TP}} + {\rm{TN}}}}{{{\text{TP}} + {\text{TN}} + {\text{FP}} + {\text{FN}}}}$$

3 $${\text{PPV}} = \frac{{{\rm{TP}}}}{{{\text{TP}} + {\text{FP}}}}$$

### Curate the clinotype—genotype association

Since we did not have genetic test information among the study cohort, we used public databases PAGER [49, 50] and REACTOME [51, 52] (pathway and metabolism only) to find genes associated with the clinotypes. PAGER is a geneset database, which integrates the most popular geneset-level databases known today (including MsigDB) and collection of phenotype-related genes from popular manual curated databases, including OMIM [53, 54], MSigDB and GeneSigDB [55]. REACTOME is one of the most well-known curated biological pathway databases known today. We removed non-biological words in each clinotype name, such as absolute value, percentage, ratio, volume, etc. and convert all names to singular form before querying. For example, with clinotypes “Basophils Percentage” and “Monocytes Absolute value”, we queried “Basophil” and “Monocyte”. After acquiring the clinotype’s related gene set, we used DAVID Gene ID conversion tool [56, 57] to map the names retrieved from REACTOME and PAGER to UniProt ID to remove potential alias names and ensure that the genes found were reviewed. After querying and filtering, we obtained 12,635 connections between 6145 genes and only 61 clinotypes, as showed in Additional file 2: Table S2.

### Find disease-phenotype and clinotype associations

#### Technical solution

Using the diagnostic information for the cohort covered in P1 subset, we found the disease-phenotype and clinotype associations with the help of student *t*-test [58] as follow. In P1, we select patients having less than 5% abnormal clinotype values and no diagnostic history into the control set. For each disease, we use the ICD10 diagnostic code to select the ‘disease’ set. Comparing between the disease and control sets with t-test, we computed the *p*-value for each clinotype. The clinotypes having significant *p*-value (less than 0.05) was considered to have significant associations with the underlying disease.

#### Performance metric and validation

To validate these associations, we compared the disease-versus-control classification performance using two types of model. For the first type of model, noted as ASS (abbreviation of association), we only use the disease’s associated tests as features for classification. For the second type of model, noted as NON (abbreviation of non-association), we only used the non-associated tests as features for classification. We trained the classification models using the Pr set and measure the performance on the Pt set, as shown in the above section. We expect that the classification metrics: area under the curve (AUC) and accuracy [59] of the ASS models should be higher than the ones in the NON model. For training classification models, we applied Random Forest [60] implemented in Weka version 3.8 [61], which was significantly successful in Google’s and Mt. Sinai’s DeepPatient [62].

### Identify subcohorts of interest by patient stratification

We used the Plotviz tool [63, 64], built upon the high-performance computing platform at Indiana University, to cluster the P2 subset patients. Deterministic Annealing Pairwise Clustering (DAPWC) algorithm [65], which focuses on highlighting the datapoint difference in high dimensional data, Plotviz significantly reduced the computational time, performed dimensionality reduction and visualize the results in 3D. To determine the number of cluster parameters (*k*) in Plotviz, we applied Silhouette index [66] (Si) to select the best number of clusters. Si closed to 1 implies appropriate clustering structure; meanwhile, Si closed to -1 implies inappropriate clustering structure, including too few and too many clusters. From multiple experiments, we choose *k* = 5 (Si = 0.793).

We proposed two option to annotate the clusters. First, we found the significant clinotypes expressing in each cluster by the ANOVA test. Clinotypes returning significant average *p*-value (less than 0.05) could be used to annotate the clusters. Second, we found which clusters *c* would over-represent a specific disease *D* using hypergeometric distribution *p*-value computed as

4 $$\mathop \sum \limits_{\tau = \kappa }^{{\text{min}}({\text{\rm K}},\eta )} \frac{{\left( {\frac{{{\text{\rm K}}!}}{{\left( {{\text{\rm K}} - \tau } \right)!\tau !}}} \right)\left( {\frac{{\left( {{\text{\rm N}} - {\text{\rm K}}} \right)!}}{{\left( {\eta - \tau } \right)!\left( {\left( {{\text{\rm N}} - {\text{\rm K}}} \right) - \left( {\eta - \tau } \right)} \right)!}}} \right)}}{{\frac{{{\text{\rm N}}!}}{{\left( {{\text{\rm N}} - \eta } \right)!\eta !}}}}$$

where ${\text{\rm N}}$ (nu) is the number of patients in P2 subset, ${\text{{\rm K}\;}}\left( {{\text{kappa}}} \right)$ is the number of patients having disease *D* diagnosis, ${\upeta }$ is the size of cluster *c* and ${\upkappa }$ is the number of patients having disease *D* in cluster *c*. The less-than-0.05 *p*-value implies that cluster *c* significantly enriches disease *D*.

## Results

In this work, we use the following acronyms:

- SVLR: support vector linear regression
- PPV: positive predictive value
- NPV: negative predictive value
- ACC: accuracy
- AUC: area under the receiver-operating characteristic curve

### Robust missing value prediction models

In tackling missing value issue, the prediction performance of SVLR is desirable for predicting values of a number of numerical clinotypes. Overall, the weighted prediction accuracy for all measurement is 0.760, the weighted average PPV is 0.488, and the weighted average NPV is 0.829. This performance is significantly higher than the random prediction, in which, due to the metric defined in the method sections, the expected random ACC/PPV/NPV would be 0.33. Additional file 3: Table S3 shows all prediction performance metrics of all clinotypes. There are three scenarios for the performance of SVLR on predicting missing clinotypes. First, Blood Platelet Hematocrit, Average Erythrocyte Volume, and Lymph Absolute Value show both high (above 0.7) PPV and accuracy. Second, Albumin, RBC Volume Distributed SD Value and Neutrophils Absolute value show average PPV (from 0.5 to 0.7) and high accuracy. Third, Lipid-related measurements, such as LDL-Cholesterol, Apolipoprotein B and Triglycerides achieve moderate PPV but moderate or low accuracy (below 0.7), except LDL cholesterol. Most of the clinotype NPVs are high, except for lipid-related measurements.

**Fig. 2:**
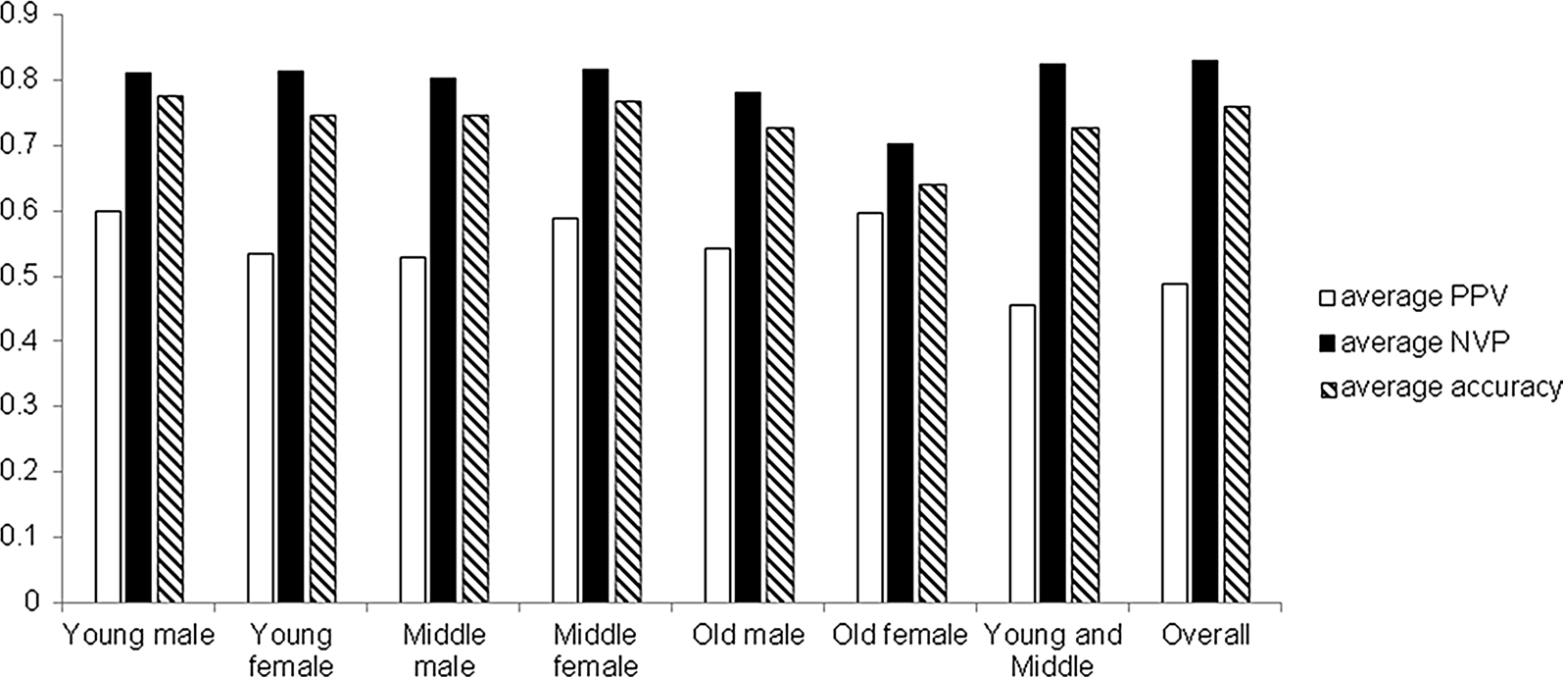
Performance of SVLR models for predicting missing values: average ACC, PPV and NPV comparison between different groups of patients (defined in the method sections)

The SVLR may not be very accurate to model clinotypes for old people. In Fig. 2, accuracy, PPV and NPV of models trained by young-age and middle-age groups are higher than the ones trained using old groups. Furthermore, the average NPV and accuracy trained by old-age groups are lower than the average NPV and accuracy using the entire dataset.

### The significant disease-phenotype-clinotype associations could potentially improve disease identification

**Fig. 3:**
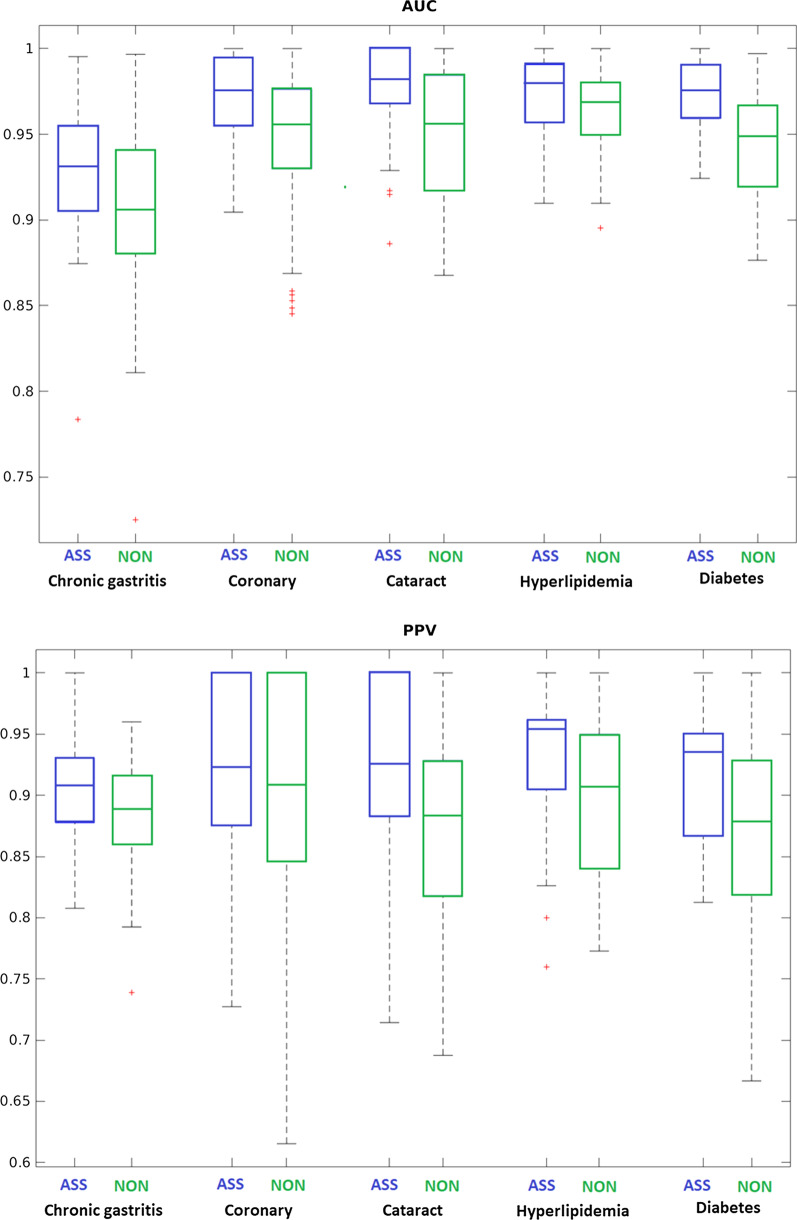
AUC/PPV comparison between two types of the disease-specific classification model: using (ASS) and not using (NON) only disease-phenotype-clinotype association

Here, we focused on the phenotype-clinotype associations of five popular chronic diseases: chronic gastritis, coronary, cataract, hyperlipidemia, and diabetes. We found 147 significant phenotype-clinotype associations (Additional file 4: Table S4). We demonstrated the top 10 significant clinotype-phenotype associations, sorted by *p*-value, in Table 3. Figure 3 shows that the classification models built upon these associations (acronym: ASS models) are completely superior to the models built without using these associations (non-association, acronym: NON models). Briefly, the ASS models only use the clinotypes that have strong associations to the diseases; while the NON models do not use these clinotypes. The details on constructing these models, from finding clinotype-phenotype associations to classification algorithms (random forest) could be found in the method section. In all diseases, the ASS models achieve higher AUC and PPV. By average, the ASS models AUC of 0.967 and PPV of 0.923; meanwhile, the NON models only achieve AUC of 0.942 and PPV of 0.886.

**Table 3:** Top 10 significant clinotype-phenotype association found in P2 dataset

Clinotype	Disease-phenotype	*p*-value
Blood crystallization	Diabetes	3.36 × 10^–18^
Blood crystallization	Coronary	1.48 × 10^–17^
Rheumatoid factor	Hypertension	1.78 × 10^–16^
Blood crystallization	Hyperlipidemia	1.47 × 10^–13^
Rheumatoid factor	Chronic gastritis	4.77 × 10^–12^
Glucose	Diabetes	1.71 × 10^–11^
Crystallization	Cataract	4.22 × 10^–11^
Rheumatoid factor	Hyperlipidemia	6.47 × 10^–9^
Blood platelet	Hyperlipidemia	6.24 × 10^–7^
Triglycerides	Hyperlipidemia	6.61 × 10^–7^

### Cohort identified by stratification of patients’ clinotype reveals potential chronic comorbidities

**Fig. 4:**
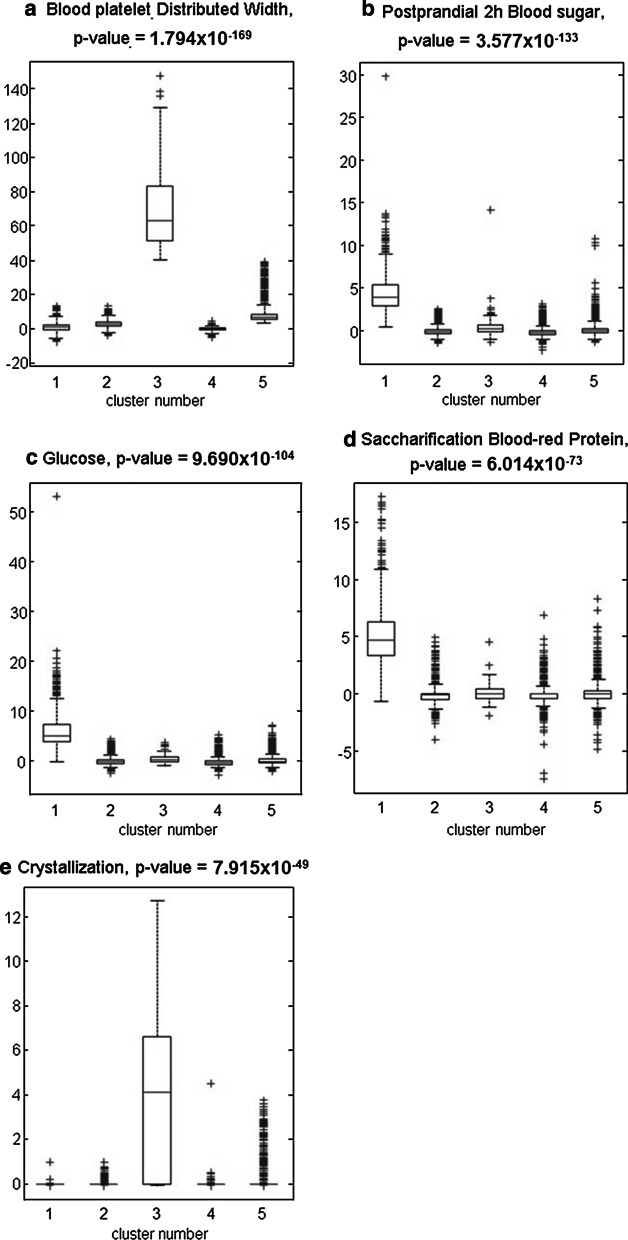
Top 5 clinotypes annotating identified subcohorts. x axis stands for the cluster index. y axis stands for the normalized clinotype values

**Fig. 5:**
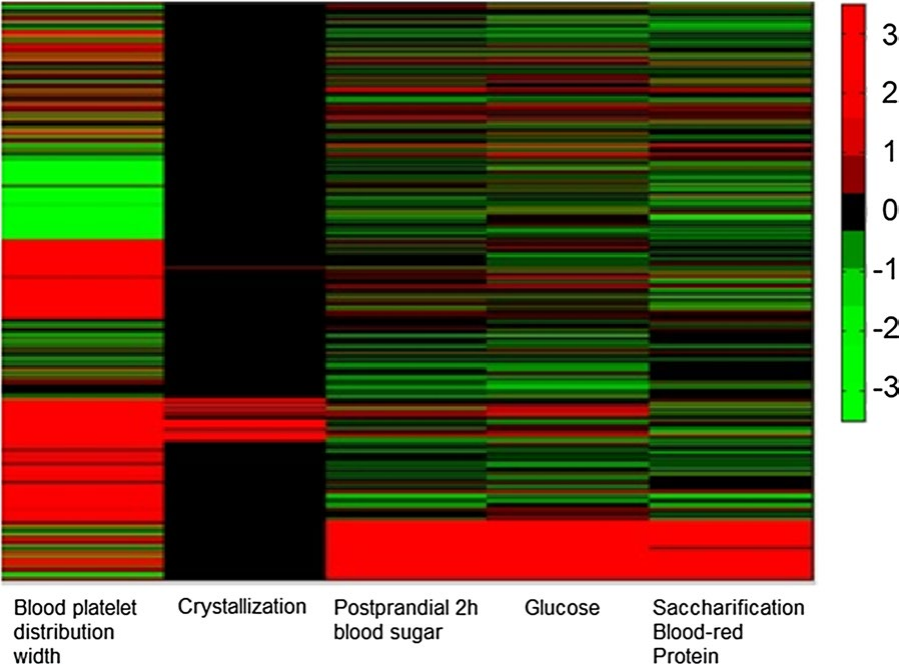
Clustering heatmap with top 5 measurements: Patients are represented by rows. The order of columns is Blood platelet Distributed Width, Crystallization, Postprandial 2h Blood sugar, Glucose, and Saccharification Blood-red Protein

For 5 subcohorts identified by Plotviz clustering, the ANOVA tests return 67 significant clinotypes (Additional file 5: Table S5) which could be used to annotate each cluster. Information for selecting the number of clusters could be found in Additional file 5. Interestingly, the unbias and domain-knowledge free clustering method (Plotviz) results in patients subgroups who have potentially similar disease phenotypes. The top 5 significant clinotypes are Blood Platelet Distributed Width (*p*-value 1.79 × 10^–169^), Postprandial 2h Blood Sugar (*p*-value 3.58 × 10^–133^), Glucose (*p*-value 9.69 × 10^–104^), Saccharification Blood Protein (*p*-value 6.01 × 10^–73^) and Crystallization (*p*-value 7.92 × 10^–49^). These top 5 clinotypes annotate two clusters. Blood platelet Distributed Width and Crystallization is higher cluster 3 containing 101 patients (Figs. 4, 5). Postprandial 2h Blood sugar, Glucose and Saccharification Blood-red Protein specify cluster 1 containing 843 patients. Additional file 6: Table S6 summarizes the disease-phenotype annotation for each cluster. These annotations could be visualized using with Plotviz (http://salsahpc.indiana.edu/plotviz/) visualization and data files in Additional file 7.

## Discussions

In this work, CPA’s machine learning technique could successfully predict the missing health clinotype values. Accurate missing-value prediction provides qualified information for supporting diagnosis and a better understanding of the patient at an individual level. In addition, Plotviz clustering technique could reveal patient subgroups who potentially share similar health issues. Validation via curation shows potential explanation about significant clinotype-clinotype associations at the gene level. This result could be used to suggest new biological research topic about the clinotype-genotype associations.

We also want to clarify the difference of “clinical modeling” concept, which our CPA framework aims for, with the “clinical information models” (CIM) defined by Moreno-Conde’s group [40]. In [40], CIM is a board concept for structural and semantic artifacts providing multiple functionalities: organizing, storing, querying, visualizing, exchanging and analyzing data. In the CPA framework, missing value prediction and clinotype-clinotype association discovery could be called analyzing data functionalities. In addition, the results from patient clustering and linking clinotypes to genomic databases could certainly lead to new clinical trials and research. Therefore, CPA could extend the CIM concept by adding the recommendation functionality, which could be very helpful for doctor and research users.

There are three main limitations of this research work. The first limitation is that the linear prediction models do not work well with patients from old-age groups. Therefore, the nonlinear methods are better-recommended to learn the clinotype-clinotypes associations the follow-up analysis from the old-age-group data. The second limitation is constructing the semantic structure among health clinotype names. Thus, we could not use standard annotation code for diseases, symptoms and other phenotypes, such as ICD10 and MeSH term to acquire better curation as in [41].

In addition, to complete the triangle among clinotype, phenotype and genotype, the CPA framework should include the following problems. First, mining clinotype-clinotype association would complete the clinotype-clinotype edge, which has not been addressed. Machine learning techniques could be reapplied in this problem. Second, linking the clinotype-clinotype and clinotype-genotype associations to the gene level would provide insights explaining the associations above. Here, integrating PheWas with better clinotype-phenotype association (from curation and natural language processing) would be a promising solution. We would solve these problems in some future work.

In addition, PPV leaves two issues for open discussion in this work. First, the weak anti-correlation between prediction accuracy and PPV leaves an issue in sampling the training set. It is expected that when we use totally random balance sampling in the training set, the distribution of predicted labels in the test set may contain less ‘normal’ label and may increase PPV. However, ‘normal’ is the major label; therefore, increasing PPV may decrease accuracy. We do not have a clear answer whether or not more advanced data sampling approaches in [42] could be a better solution due to the missing value. Second, although the average PPV achieved in this work is moderate (PPV), we argue that it is a reportable outcome. In this study, the ‘positive’ class stands for abnormal measurement value (either high or low), which is often the minor class in health data. In addition, our definition for true positive (see method section of setup metrics for prediction performance) only allows the predicted label and the true label as either ‘high’ or ‘low’. In other words, if the predicted is ‘low’ but the true label is ‘high’ and vice versa, we still consider this case as false positive although both the predicted label and the true label are not ‘normal’. With this definition, the expected random PPV is 0.33, much less than the average PPV we achieved. Our plausible results in clinotype-clinotype association discovery and patient clustering, which directly use clinotype missing value prediction, show that the discovery is still solid with the PPV above. However, we believe that the discovery could be improved if we apply other techniques with higher PPV.

## Conclusions

By CPA framework, we showed how utilizing clinical test results information (clinotype) could further support precision medicine. The proposed problems and solutions with clinotypes demonstrate that clinotype could potentially be an independent area but associating with the well-known genotype–phenotype association studies. Machine learning techniques play a key role in this pioneering work. It could lay out the general ideas from which the future techniques could improve the solution for each problem proposed in this work.

## Supplementary Information

**Additional file 1.** Count of patients diagnosed with each disease (identified by Chinese ICD10).

**Additional file 2.** Curated associations between clinotype and genotype.

**Additional file 3.** Missing value models performance in all clinotypes.

**Additional file 4.** List of significant clinotype - phenotype (disease) associations, with p-value < 0.05.

**Additional file 5.** ANOVA test result for each clinotype when using to annotate the disease cohorts.

**Additional file 6.** Hypergeometric enrichment test result when annotating patient-clusters by phenotype.

**Additional file 7.** Five cluster visualization using PlotViz software.

## Data Availability

The original datasets are not included in this work. Researchers interested in using the dataset should contact Chuandi Pan or Jake Chen for further details and permission.
